# Associations of multimorbidity and patient‐reported experiences of care with conservative management among elderly patients with localized prostate cancer

**DOI:** 10.1002/cam4.3274

**Published:** 2020-07-06

**Authors:** Ryan M. Fiano, Gregory S. Merrick, Kim E. Innes, Malcolm D. Mattes, Traci J. LeMasters, Chan Shen, Usha Sambamoorthi

**Affiliations:** ^1^ Wheeling Hospital Urologic Research Institute Schiffler Cancer Center Wheeling WV USA; ^2^ West Virginia Clinical and Translational Science Institute Morgantown WV USA; ^3^ Department of Epidemiology West Virginia University Morgantown WV USA; ^4^ Department of Radiation Oncology Rutgers Cancer Institute of New Jersey New Brunswick NJ USA; ^5^ West Virginia University School of Pharmacy Pharmaceutical Systems & Policy Morgantown WV USA; ^6^ Penn State Health Milton S Hershey Medical Center Hershey PA USA

**Keywords:** active surveillance, conservative management, multimorbidity, patient‐centered care, prostate cancer

## Abstract

**Background:**

Many elderly localized prostate cancer patients could benefit from conservative management (CM). This retrospective cohort study examined the associations of patient‐reported access to care and multimorbidity on CM use patterns among Medicare Fee‐for‐Service (FFS) beneficiaries with localized prostate cancer.

**Methods:**

We used linked Surveillance, Epidemiology, and End Results cancer Registry, Medicare Claims, and the Medicare Consumer Assessment of Healthcare Providers and Systems (MCAHPS) survey files. We identified FFS Medicare Beneficiaries (age ≥ 66; continuous enrollment in Parts A & B) with incident localized prostate cancer from 2003 to 2013 and a completed MCAHPS survey measuring patient‐reported experiences of care within 24 months after diagnosis (n = 496). We used multivariable models to examine MCAHPS measures (getting needed care, timeliness of care, and doctor communication) and multimorbidity on CM use.

**Results:**

Localized prostate cancer patients with multimorbidity were less likely to use CM (adjusted odds ratio (AOR)=0.42 (0.27‐ 0.66), *P* < .001); those with higher scores on timeliness of care (AOR = 1.21 (1.09, 1.35), *P* < .001), higher education attainment (3.21 = AOR (1.50,6.89), *P* = .003), and impaired mental health status (4.32 = AOR (1.86, 10.1) *P* < .001) were more likely to use CM.

**Conclusion(s):**

Patient‐reported experience with timely care was significantly and positively associated with CM use. Multimorbidity was significantly and inversely associated with CM use. Addressing specific modifiable barriers to timely care along the cancer continuum for elderly localized prostate cancer patients with limited life expectancy could reduce the adverse effects of overtreatment on health outcomes and costs.

## Graphical abstract

Timeliness of care was significantly and positively associated, and multimorbidity was significantly and inversely associated with conservative management patterns among localized prostate cancer patients. Addressing specific modifiable barriers to timely care along the cancer continuum could help reduce overtreatment of elderly localized prostate cancer patients with multimorbidity and limited life expectancy.

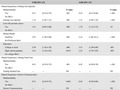

## 1 INTRODUCTION

Conservative management (CM) has emerged as a preferred disease management approach for many older adults with localized prostate cancer.^1^ CM use in patients with low‐risk localized prostate cancer or limited life expectancy is supported by high‐level evidence.^2^ CM includes protocols for low‐ or intermediate‐risk prostate cancer, such as follow‐up biopsies and PSA testing, or “watchful waiting” for patients with less than 5 years of life expectancy. Use of CM among patients with low‐risk prostate cancer or limited life expectancy improves health‐related quality of life (ie, urinary, bowel, and/or erectile dysfunction) and could reduce excessive annual health‐care costs of overtreatment by $1.2 billion.^3^, ^4^

CM decisions are complex as 60% of older adults (age > 65 years) with localized prostate cancer have preexisting multimorbidity.^5^, ^6^ Multimorbidity affects life expectancy^5^ and more than 50% of patients with multimorbidity seek care from three or more health‐care specialists.^7^ Patients with multimorbidity and cancer may be vulnerable to poor quality of cancer care and have prompted greater attention in measuring, monitoring, and incentivizing patient‐centered care.^8^ Measures of patient‐centered care, such as patient‐reported experiences with care include domains of physician communication, timely care, and perceptions of getting needed care, are increasingly used as quality measures by health plans, medical groups, and physician practices. Positive patient‐reported experience scores are associated with adherence to medical advice, improved clinical outcomes, and lower utilization of unnecessary health‐care services^9^, ^10^ such as overtreatment of low‐risk localized prostate cancer.

Patient‐reported experiences may differ by multimorbidity status, which may further complicate or facilitate treatment choices for low‐risk prostate cancer.^11^ Identifying specific measures of patient‐reported experiences that facilitate CM use among patients with incident localized prostate cancer and multimorbidity is needed to promote evidence‐based cancer care.^12^ For example, in colorectal cancer populations, patient‐reported experiences with perceived timely care are associated with evidenced‐based follow‐up.^13^ Understanding the relationship between patient‐reported experiences of care on CM use can inform patient‐centered care approaches to improve adoption of CM use, thereby reducing the adverse effects of overtreatment among older patients with multimorbidity and localized prostate cancer.

Despite the importance of patient‐reported experiences, CM studies primarily focus on disease characteristics, clinical, and sociodemographic factors.^1^, ^14^, ^15^, ^16^ To date, no studies have investigated the impact of patient‐reported experiences on CM use among medically complex patients with localized prostate cancer. Therefore, the primary objective of this study is to examine the associations of multimorbidity and patient‐reported experiences on CM use among Fee‐for‐Service (FFS) Medicare beneficiaries with localized prostate cancer using Consumer Assessment of Healthcare Providers and Systems (MCAHPS®) patient surveys and Medicare claims linkages.

## 2 METHODS

**Figure 1:**
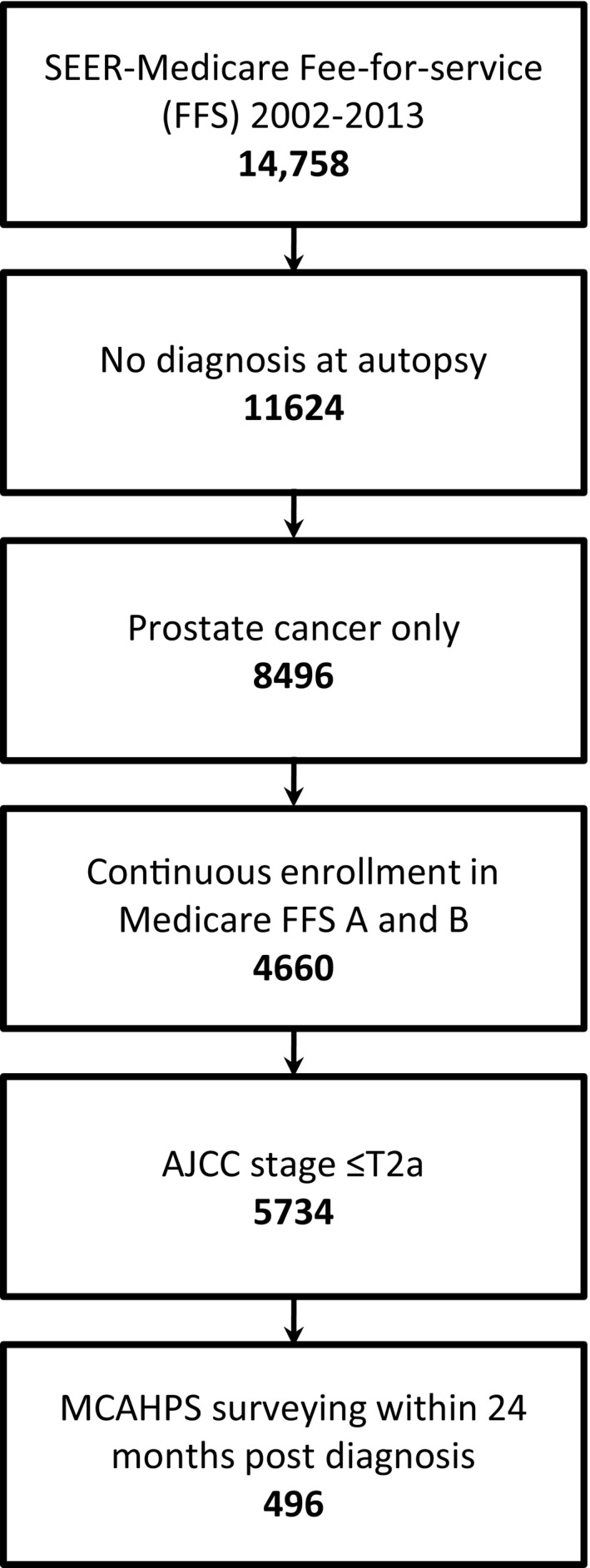
Cohort Selection and Exclusion

The study cohort included men diagnosed with localized prostate cancer defined as American Joint Committee on Cancer stage T2a or less,^2^ aged 66 or older, with continuous enrollment in FFS Medicare Parts A and B throughout the study period (Figure 1).

Date of incident localized prostate cancer diagnosis was used as an index date and 12 months before diagnosis was used as the baseline period. During the baseline period, we identified multimorbidity using Medicare claims and calculated life expectancy estimates.

We also defined the “CM measurement” period as 12 months after diagnosis. During this period, we identified CM based on validated methods for claims data.^17^

As MCAHPS surveys can be administered at varying points during the postdiagnosis period, we followed individuals for an additional period of 12 months. Thus, our follow‐up period consisted of 24 months after incident localized prostate cancer diagnosis.

To account for varying months from diagnosis to survey administration, we included time from diagnosis to survey as one of the independent variables in the models. However, as this variable was not significant and did not affect our main results, we did not include time from diagnosis to survey administration variable in the final model. As a sensitivity analysis, we also estimated CM use during 24 months after diagnosis (Table S2).

### 2.1 Data sources

The Surveillance, Epidemiology, and End Results (SEER) cancer registry contains tumor and demographic information for patients diagnosed with cancer while residing in a SEER region. We derived Medicare eligibility from the SEER data (Figure 1). We extracted FFS Medicare claims from Medicare Provider Analysis and Review (MEDPAR), Carrier Claims, Outpatient Claims, Home Health Agency, and Durable Medical Equipment files.

Medicare Consumer Assessment of Healthcare Providers & Systems (MCAHPS®) surveys, administered by the CMS, use standardized and validated questionnaires to collect information on patient‐reported experiences with health‐care providers.^18^ MCAHPS collection methodologies use a weighted probability sampling procedure covering all the 50 US states, DC, and Puerto Rico, which are then linked to SEER patients.^18^, ^19^

Area Health Resource File (AHRF) files were linked via MEDPAR FIPS state and county codes and were used to calculate radiation oncologist and urologist densities.^20^ Census files were linked to calculate county‐level median income quartiles.

### 2.2 Dependent Variable

We operationalized CM use as the absence of curative treatment within 12 months after incident localized prostate cancer. Treatment was identified using International Classification of Diagnosis 9th edition (ICD9), ICD9 procedure codes, and Healthcare Common Procedure Coding System (HCPCS) codes from FFS Medicare claims (Table S3).^2^, ^15^, ^17^

### 2.3 Key independent variables

The *multimorbidity* framework developed by the United States Department of Health and Human Services for guiding programs, practice, and policy guided the selection of chronic conditions as follows: arthritis, asthma, coronary artery disease, congestive heart failure, chronic kidney disease, chronic obstructive pulmonary disease, cardiac arrhythmias, acute myocardial infarction, dementia, diabetes, depression, hepatitis, hyperlipidemia, hypertension, human immunodeficiency virus, osteoporosis, substance abuse, schizophrenia, stroke, anemia, and lower limb fracture.^21^ The most common definition of multimorbidity is the concurrent presence of two or more conditions in the same individual.^22^ We defined multimorbidity as the presence of three or more conditions in the same individuals as older men diagnosed with prostate cancer at age 65 or higher are at high risk for competing risk mortality. For example, among men with three or more comorbid conditions, aged 61‐74 and 75 years or older, 10‐year other cause mortality is 40% and 71%, respectively.^23^

*Prostate cancer comorbidity index* (PCCI), a weighted comorbidity index validated prostate cancer patient populations, was used to predict 5‐ and 10‐year life expectancy in prostate cancer patient populations.^24^ PCCI was calculated during the baseline period to estimate 5‐ and 10‐year life expectancy. PCCI was categorized into three groups: 0–8 (>10‐year life expectancy); 9 to 13 (5‐ to 10‐year life expectance); and > 13 (<5‐year life expectancy). In all models, PCCI total 0‐8 was used as the reference group.

Published research in prostate cancer patients often uses Charlson Comorbidity Index (CCI); therefore, we conducted a supplemental analysis using CCI. In these analyses, CCI scores were dichotomized with “0‐1” as the reference group.^25^

We included three MCAHPS composite measures—“getting needed care,” “getting care quickly,” and “doctor communication”—which rates the ability to get needed appointments, timeliness of care when care is needed, and how well the physician communicated.^18^ Patients report experiences with health‐care access in the last 6 months.^18^ MCAHPS surveys have been extensively validated for measuring patient‐reported access to care and are commonly used for quality improvement as well as value‐based purchasing initiatives.^18^ MCAHPS are based on a 0‐100 scale with 0 representing the lowest and 100 representing the highest score; we examined the effect of 10 unit changes in composite items on the dependent variable.

**Figure 2:**
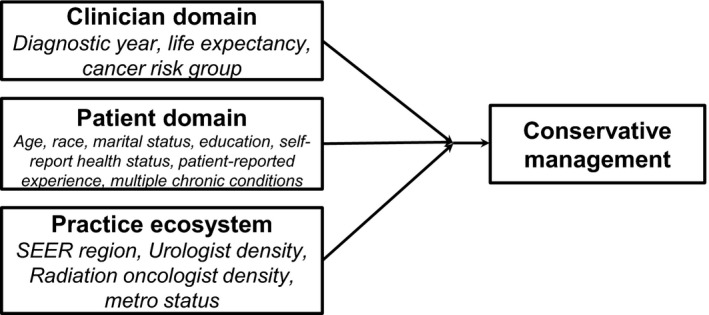
Competing Demands Framework

Management of preexisting multimorbidity and shared prostate cancer treatment decision‐making requires the use of limited resources (ie, time to manage chronic conditions and availability of health‐care professionals and resources). Therefore, for other independent variables, the competing demands model was used to conceptualize factors known to affect localized prostate cancer treatment selection within clinician, patient, and practice ecosystem domains.^15^, ^17^, ^26^ (Figure 2).

Multivariable models were adjusted with independent variables: diagnosis year group (2003‐2009 and 2010‐2013), low‐risk prostate cancer (operationalized as Gleason Score ≤ 6 and PSA test ≤ 10 ng/mL or* *Gleason Score>=7 or PSA> = 10 ng/mL), self‐reported general and mental health status, education‐level, zip‐code income quartiles, and county‐level quartiles of urologists and radiation oncologists per 10,000 persons over age 65.

Our analyses include several case‐mix adjustment variables such as age, education, general health status, mental health status, income level, and race. Secondary analyses using additional recommended case‐mix adjustment variables, such as dual‐eligible Medicaid respondents, “proxy” survey completion, and time from cancer diagnosis to survey completion, did not significantly improve model specification.^27^

To assess the potential influence of missing data, we examined missing data patterns using covariate‐dependent missingness methods.^28^ Mean values were imputed to independent variables of interest. For categorical variables, including general and mental health status, missing data indicators were created and included as a separate category in the regression models.

Chi‐square tests and t tests were used to identify significant group differences in CM use by categorical variables. Multivariable models were fit using separate unadjusted and adjusted logistic regressions to identify independent and interactive associations of multimorbidity and patient care experiences on CM use. All statistical tests were two‐sided with a 5% Type I error rate and were completed in STATA (StataCorp, College Station, TX).

## 3 RESULTS

The study cohort was predominantly non‐Hispanic, whites (84.5%). The median age at diagnosis was 72.8 years and did not differ by year of diagnosis (2003‐2009:73.6 = M, 5.38; 2010‐2013:73.6 = M, SD = 5.14). Average composite scores for doctor communication, getting needed care, and getting care quickly were 91.0 (SD = 12.2), 88.6 (SD = 15.6), and 70.8 (SD = 21.7), respectively.

Overall 33.5% used CM, defined as no curative treatment within 12 months of incident localized prostate cancer diagnosis. Use of CM was only marginally higher in men with low‐risk relative to those with higher‐risk disease (≤ cT2a and PSA>= 10 ng/mL or Gleason Score > 6) (38.7% vs 30.9%, respectively, *P* = .08) (Table 1). High‐school graduation, college education, low‐risk prostate cancer diagnosis, and mental health status were significantly more frequent among patients using CM (Table 1). CM use by localized prostate cancer patients with higher‐risk disease was 30.9%. Higher‐risk disease was significantly more common among age groups 75+ (75.9%) vs 66‐74 (62.4%) (*P* = .002) and significant differences by patients with multimorbidity 24.6% (n = 45) vs those without multimorbidity 38.7% (n = 58), (Χ^2^ = 7.65, *P* = .006) were observed.

**Table 1:** Patient Characteristics by Conservative Management among Fee‐for‐Service Medicare Beneficiaries with Incident Localized Prostate Cancer using Linked SEER Cancer Registry with MCAHPS, 2002‐2013 (n = 496)

	CM		No CM		Χ^2^	*P*‐value
	N	%	N	%		
ALL	166	33.5	330	66.5		
Age in Years					1.32	.250
66‐74	102	31.7	220	68.3		
75+	64	36.8	110	63.2		
Race					0.07	.964
White	140	33.4	279	66.6		
Black	15	32.6	31	67.4		
Other	11	35.5	20	64.5		
Marital Status					0.2	.905
Married	115	32.9	235	67.1		
Unmarried	22	34.9	41	65.1		
Unknown	29	34.9	54	65.1		
Income quartiles					0.54	.909
First	38	33.3	76	66.7		
Second	39	33.6	77	66.4		
Third	38	31.1	84	68.9		
Four	51	35.4	93	64.6		
Education					8.11	**.017**
College or more	100	36.6	173	63.4		
High‐School Grad.	42	35.9	75	64.1		
No High‐School Grad.	13	18.8	56	81.2		
General health status					3.32	0.19
Excellent/Very Good	59	35.5	107	64.5		
Good	54	28.6	135	71.4		
Fair/Poor	47	37.6	78	62.4		
Mental health status					11.3	**.004**
Excellent/Very Good	104	31.6	225	68.4		
Good	34	30.1	79	69.9		
Fair/Poor	22	57.9	16	42.1		
Urologist density					4.99	.173
0 to 1.41	41	33.1	83	66.9		
1.41 to 2.49	33	26.6	91	73.4		
2.5 to 3.46	51	39.8	77	60.2		
3.47 to 10.2	41	34.2	79	65.8		
Radiation oncologist density					5.43	.143
0 to 0.44	37	29.8	87	70.2		
0.45 to 1.07	41	33.1	83	66.9		
1.07 to 1.49	52	41.6	73	58.4		
1.51 to 5.35	36	29.3	87	70.7		
SEER region					5.09	.166
Northeast	33	35.5	60	64.5		
South	35	30.4	80	69.6		
North‐central	13	22.4	45	77.6		
West	85	37	145	63.0		
Metro Status					0.71	.401
Metro	138	34.3	264	65.7		
Nonmetro	28	29.8	66	70.2		
Diagnosis Year					0.44	.509
2002–2007	96	32.3	201	67.7		
2008–2013	70	35.2	129	64.8		
Low‐risk prostate cancer					2.93	.087
Yes	63	38.7	100	61.3		
No	103	30.9	230	69.1		

Note

Bold values denote statistical significance at the *P*‐value < .05 level.

Based on 496 older (age ≥ 66 years) Fee‐for‐Service Medicare beneficiaries, with continuous enrollment in Medicare Part A & Part B, diagnosed with incident localized prostate cancer between 2003 and 2013.

Abbreviations: CM, Conservative management; MCAHPS, Medicare Claims and the Medicare Consumer Assessment of Healthcare Providers and System surveys; SEER, Surveillance, Epidemiology, and End Results cancer Registry.

In our study cohort, 57.2% had multimorbidity. Patients 75 years or older were significantly more likely to have multimorbidity than those aged 66‐74 years (64.4% vs 53.4%). Blacks had a higher percentage of multimorbidity as compared to whites (76.1% vs 53.9%). Patients with multimorbidity using treatment (n = 207) did not differ significantly by patient, clinician, or practice ecosystem factors except for mental health status of excellent/very good (74.6%) and good (77.5%) vs patients using CM (*P* = .031). Patients with multimorbidity and higher‐risk disease (n = 183) significantly more frequently used treatment if aged 66‐74 (82.5%) (*P* = .011). Average composite scores for doctor communication, getting needed care, and getting care quickly did not differ by multimorbidity status. CM use was significantly lower in men with vs without multimorbidity (27.1% vs 72.9%, respectively, *P* < .001).

Average getting care quickly composite scores (ie, timely care) were higher for those with CM use as compared to those without CM use (Table 2). CM use significantly differed by PCCI categories, with lower percentages among groups with less than 10 (27.0%) and 5 (27.0%) vs more than 10 (38.9%) years of life expectancy (Χ^2^ = 7.82, *P* = .020) (Table 2).

**Table 2:** Multimorbidity and Patient Experiences by Conservative Management among Fee‐for‐Service Medicare Beneficiaries with Incident Localized Prostate Cancer using Linked SEER Cancer Registry with MCAHPS, 2002‐2013

	**CM**		**No CM**		Χ^2^	*P*‐value
	N	%	N	%		
	**166**	**33.5**	**330**	**66.5**		
Multimorbidity					12.1	<.001
Yes	77	27.1	207	72.9		
No	89	42.0	123	58.0		
PCCI					7.82	.020
< 5 years life expectancy	20	27.0	54	73.0		
5‐10 years life expectancy	41	27.0	111	73.0		
>10 years life expectancy	105	38.9	165	61.1		

	Mean	SE	Mean	SE	t‐Value	*P*‐value
Getting Needed Care	87.1	1.33	89.3	0.81	1.48	NS
Getting Care Quickly	75.0	1.48	68.7	1.25	−3.06	.002
Doctor/Patient Communication	89.8	1.05	91.6	0.63	1.61	NS

Note

Based on 496 older (age ≥ 66 years) Fee‐for‐Service Medicare beneficiaries, with continuous enrollment in Medicare Part A & Part B, diagnosed with incident localized prostate cancer between 2003 and 2013.

Abbreviations: CM, Conservative management; MCAHPS, Medicare Consumer Assessment of Healthcare Providers and System surveys; N.S, Not Significant; PCCI, Prostate Cancer Comorbidity Index; SEER, Surveillance, Epidemiology, and End Results cancer Registry.

PCCI life expectancy groups did not statistically differ by CM use. Higher‐risk patients reporting fair or poor mental health status (62.1%; *P* = .002) vs excellent mental health status, and college education (33%) or high‐school graduates (37.5%) vs no high‐school graduation (13.7%), significantly used CM more frequently. Getting care quickly composite scores were significantly higher among higher‐risk patients (n = 333) using CM (M = 75.8) vs curative treatment (M = 69.8), (t=−2.43, CI 95%= 69.3, 73.9, *P* = .016).

Multimorbidity was significantly and inversely related to CM use in unadjusted logistic regression analyses (odds ratios (OR) = 0.55; 95% CI = 0.35, 0.75). Adjustment for other factors, including timeliness of care, further strengthened this association (adjusted OR (AOR) = 0.42, CI 0.27‐ 0.66) (Table 3); additional models adjusting for other patient experience domains or CCI were not significant. Getting care quickly showed a significant, positive association with CM use in both the unadjusted analyses (OR = 1.15; 95% CI = 1.05, 1.27) and the fully adjusted models (AOR = 1.21; 95% CI = 1.09, 1.34). In models including PCCI life expectancy categories, less than 10‐ and 5‐year life expectancy were inversely associated with CM use (Table S1).

**Table 3:** Multivariable Analysis of Timeliness of Care, Multimorbidity, and Factors Associated with Conservative Management Use vs Treatment among Fee‐for‐Service Medicare Beneficiaries with Incident Localized Prostate Cancer using Linked SEER Cancer Registry with MCAHPS, 2002‐2013 (n = 496)

	UOR [95% CI]			AOR [95% CI]		
Patient Experience: Getting Care Quickly						
Multimorbidity			***P*‐value**			***P*‐value**
Yes	0.51	[0.35‐0.75]	.001	0.42	[0.27‐0.66]	<.001
No (Ref.)						
Getting Care Quickly	1.15	[1.05‐1.27]	.003	1.21	[1.09‐1.35]	<.001
Low‐risk prostate cancer						
Yes	1.41	[0.95‐2.08]	.088	1.76	[1.14‐2.72]	.01
No (Ref.)						
Mental Health						
Fair/Poor	2.97	[1.50‐5.90]	.002	4.32	[1.86‐10.1]	<.001
Ex/VG/Good (Ref)						
Education						
College or more	2.49	[1.30‐4.78]	.006	3.21	[1.50‐6.89]	.003
High‐school graduate	2.41	[1.18‐4.92]	0.015	3.53	[1.59‐7.83]	.002
No college (Ref.)						
Patient Experience: Getting Need Care						
Multimorbidity						
Yes	0.51	[0.35‐0.75]	.001	0.45	[0.30‐0.70]	<.001
No (Ref.)						
Getting Needed Care	—	—	NS	—	—	NS
Patient Experience: Doctor Communication						
Multimorbidity						
Yes	0.51	[0.35‐0.75]	0.001	0.45	[0.29‐0.68]	<.001
No (Ref.)						
Doctor Communication	—	—	NS	—	—	NS

Note

Based on 496 older (age ≥ 66 years) Fee‐for‐Service Medicare beneficiaries, with continuous enrollment in Medicare Part A & Part B, diagnosed with incident localized prostate cancer between 2003 and 2013. Adjusted for age, race, marital status, income, education, health status, urologist density, radiation oncologist density, SEER region, geography, diagnostic year, and low‐risk prostate cancer.

Abbreviations: AOR, Adjusted Odds Ratio; CI, Confidence interval; CM, Conservative management; MCAHPS, Medicare Consumer Assessment of Healthcare Providers and System surveys; NS, Not significant; Ref., Reference group; SEER, Surveillance, Epidemiology, and End Results cancer Registry; UOR, Unadjusted Odds Ratio; Statistically significant results displayed.

CM use was also significantly and positively associated with fair/poor mental health status, low‐risk prostate cancer diagnosis, college education or more, and high‐school graduation in all adjusted models (Table 3; Tables S1 and S2). We found no evidence for a modifying effect of patient‐experience variables, multimorbidity, PCCI, or other independent variables on the observed associations.

## 4 DISCUSSION

In this study, we assessed the independent associations of multimorbidity and patient‐reported experiences with care on CM use among older men with localized prostate cancer. Despite proven benefits of CM, one in three (33.5%) of all men with localized prostate cancer, and only two in five (41%) men over the age of 75 years, used CM. Our estimates of CM use among patients with localized prostate cancer are lower than those reported in recent investigations using SEER‐Medicare data (42.1% in 2015)^1^ but higher than reported in an investigation of Michigan Medicare beneficiaries (22.3% in 2014).^17^ We speculate that these differences could be due to variation in study population characteristics (ours included prostate cancer patients from many regions of the US) and geographic practice patterns.^29^, ^30^, ^31^

Multimorbidity and life expectancy are critical components of patient counseling after a localized prostate cancer diagnosis as many older men do not live long enough to benefit from treatment. Patients with low or favorable intermediate‐risk disease or higher‐risk disease with limited life expectancy could avoid significant urinary, erectile, and rectal treatment morbidities without increasing the risk of prostate cancer‐specific mortality with CM.^2^, ^32^ However, in our study, men with multimorbidity were significantly less likely to use CM compared to those without multimorbidity after controlling for age, low‐risk prostate cancer, and other sociodemographic variables. We speculate that men with multimorbidity and low‐risk cancer may not opt for treatment because they may have a preference for immediate cure (ie, “take care of it”) ^33^ and may not want to add one more condition that requires long‐term management. Furthermore, men with multimorbidity may fear nontreatment regret,^34^ emotional distress,^35^ and anxiety.^36^ Strong multidisciplinary management strategies, including significant psychological support from primary care physicians and specialists (ie, urologist and/or medical and radiation oncologists), are needed to mitigate decisional conflict^37^ and therefore facilitate CM use.^14^

In adjusted models, including validated life expectancy measures for prostate cancer survivors, patients meeting evidence‐based criteria^2^ for CM were 58% less likely to use CM based on life expectancy alone (ie, less than 5 years). Previous studies using CCI report both positive and negative relationships between comorbidity burden and CM use in Medicare FFS populations.^38^, ^39^, ^40^ In a supplemental analysis in this study, CCI was not significantly associated with CM use. Taken together, these findings suggest that clinical and population differences in comorbidity definitions are likely to account for mixed findings in several previous investigations.^41^ By defining multimorbidity using a list of conditions prioritized by policy makers in the US,^21^ our study made a unique contribution to this field. However, as pointed out by a systematic review that current life expectancy prediction tools lack both practical and theoretical utility,^42^ comorbidity measures that can be easily operationalized in a clinical setting are needed. Recently, age‐adjusted indexes, such as the PCCI used in our study, were developed to provide life expectancy estimates in patients with prostate cancer.^24^ Certain types (cardiovascular disease) and combinations (cardiometabolic and respiratory)^43^ of chronic conditions are associated with treatment regardless of patient, clinician, and health‐care ecosystem factors. Additional research is needed to understand the relationship between more precise estimates of life expectancy and multimorbidity on CM use in FFS Medicare populations.

In our study, patient‐reported experiences, specifically timeliness of care, were positively associated with CM use. Patients with higher timeliness of care scores were significantly more likely to use CM after adjusting for demographic, clinical, socioeconomic, and health‐care system factors. Timely access to care for localized prostate cancer patients is not limited to initial diagnosis of prostate cancer, but the opportunity and ease by which a patient is able to utilize needed services along the continuum of care throughout survivorship.^44^Choices for elderly localized prostate cancer patients involve selecting curative and noncurative treatments with trade‐offs in efficacy, potential adverse quality of life effects, and competing risk mortality. MCAHPS timeliness of care domains, such as perceived barriers to appointment scheduling, are fundamental to shared decision‐making among multiple health‐care providers that significantly influence localized prostate cancer treatment choice.^45^, ^46^, ^47^ We speculate that patients with higher timely care ratings may choose CM because they may have a favorable perception of health‐care system capacity to provide services once a need is detected.

Our study findings have important policy implications. Currently, no value‐based mechanisms exist to support the use of CM in Medicare FFS populations. Existing literature also suggest that CM use in FFS system varies among physician practices by 5.1%‐71.2%.^31^ Emerging oncology care models by CMS may need to incorporate risk‐adjusted CM use as a quality indicator along the cancer care continuum ^48^ to promote CM use among men with localized prostate cancer. Recently, the NCCN Quality and Outcomes committee identified significant gaps in evaluating high‐quality cancer care with patient experience measures and evidence‐based practice.^49^ More research is needed to identify specific barriers to timely care and how validated patient‐reported experience measures can be used to support evidence‐based management of localized prostate cancer patients in oncology care models.

We also observed that elements of social determinants, such as education, were associated with CM use. Although educational attainment may not be modifiable among older adults, initiatives such as “health in all policies” by World Health Organization and the Centers for Disease and Prevention Control “integrate and articulated health considerations” into community health policy.^50^ These experts concluded that social, economic, and physical environments have a significant impact on the health of an individual and these effects should be considered in the development of all public policies and programs.

### 4.1 Strengths and limitations

The SEER‐CAHPS data linkage is a robust and unique resource that provides an ideal opportunity to study patient‐centered care delivery of contemporary treatment patterns among patients with localized prostate cancer and multimorbidity. We build on previous findings by including validated estimations of life expectancy and definitions of multimorbidity to access the impact of comorbid conditions on patterns of contemporary treatment options for older localized prostate cancer patients.

Our study results must be interpreted with important limitations. MCAHPS surveys request patient‐reported experiences with care “in the last 6 months”.^18^ Due to relatively small sample size, we included surveys completed within 6 months after localized prostate cancer diagnosis which may overlap with the baseline period. However, our results were robust to multivariable adjustments for time between cancer diagnosis and survey completion. Due to MCAHPS survey administration processes and collection, we cannot directly attribute MCAHPS composite ratings to physician specialty or the prostate cancer diagnosis; instead, our results are generalizable to the entire patient experience after diagnosis which may include multiple care providers for multiple conditions. The study sample comprised of predominantly non‐Hispanic white, urban adults, potentially limiting generalizability to ethnic minorities, rural, or other populations. Our study was restricted to Medicare FFS enrollees 65 years or older and may not be generalizable to younger adults or individuals on private insurance. Lastly, due to sample size limitations, we were unable to analyze the relationship of individual chronic conditions with CM use.

## 5 CONCLUSIONS

Our results highlight the effect of patient‐reported experiences, multimorbidity, and life expectancy on CM use among older men with localized prostate cancer. While factors such as multimorbidity and life expectancy are critical clinical components that may affect the choice of CM vs over treatment, our study highlights the role of nonclinical factors, specifically patient‐reported experiences with care on treatment of localized prostate cancer. Our findings support a “population health‐based” oncology care model in which both clinical and nonclinical factors, such as patient‐reported experiences, are integrated to promote CM use and avoid overtreatment among older men with localized prostate cancer.

## 6 PROTECTION OF HUMAN SUBJECTS

This study was conducted after approval by the institutional review board at West Virginia University and in accordance with an assurance filed with and approved by the US Department of Health and Human Services.

## CONFLICT OF INTEREST

The authors report no conflict of interests.

## AUTHOR CONTRIBUTIONS

Ryan Fiano: conceptualization, data curation, formal analysis, methodology, writing ‐ original draft, and writing ‐ review and editing. Gregory S. Merrick: writing ‐ review and editing. Kim E Innes: writing ‐ review and editing. Malcolm Mattes: writing ‐ review and editing. Traci Lemasters: writing ‐ review and editing. Chan Shen: writing ‐ review and editing. Usha Sambamoorthi: writing ‐ review and editing, data curation, formal analysis, and methodology.

## Supporting information

Table S1

Table S2

Table S3

## Data Availability

The NCI's Surveillance, Epidemiology, and End Results (SEER) cancer registry data and the Centers for Medicare & Medicaid Services' (CMS) Medicare Consumer Assessment of Healthcare Providers and Systems (CAHPS®) survey linkage were utilized in the present study (https://healthcaredelivery.cancer.gov/seer‐cahps/).

## References

[1] Mahal BA , Butler S , Franco I , et al. Use of active surveillance or watchful waiting for low‐risk prostate cancer and management trends across risk groups in the United States, 2010–2015. JAMA. 2019;321(7):704‐706. 10.1001/jama.2018.19941 30743264 PMC6439610

[2] National Comprehensive Cancer Network . NCCN Clinical Practice Guidelines in Oncology (NCCN Guidelines) Prostate Cancer Version 2.2020. https://www.nccn.org/professionals/physician_gls/pdf/prostate.pdf. Published May 2020. Accessed May 25, 2020.

[3] Chen RC , Basak R , Meyer A‐M , et al. Association between choice of radical prostatectomy, external beam radiotherapy, brachytherapy, or active surveillance and patient‐reported quality of life among men with localized prostate cancer. JAMA. 2017;317(11):1141‐1150. 10.1001/jama.2017.1652 28324092 PMC6284802

[4] Aizer AA , Gu X , Chen M‐H , et al. Cost implications and complications of overtreatment of low‐risk prostate cancer in the United States. J Natl Compr Canc Netw. 2015;13(1):61‐68. 10.6004/jnccn.2015.0009 25583770

[5] DuGoff EH , Canudas‐Romo V , Buttorff C , Leff B , Anderson GF . Multiple chronic conditions and life expectancy: a life table analysis. Med Care. 2014;52(8):688‐694. 10.1097/MLR.0000000000000166 25023914

[6] National Cancer Institute . U.S. Population Data 1969–2017 ‐ SEER Population Data. Surveillance, Epidemiology, and End Results (SEER) Program Populations (1969–2017) (www.seer.cancer.gov/popdata), National Cancer Institute, DCCPS, Surveillance Research Program. 2018 https://seer.cancer.gov/popdata/. Published December 2018. Accessed September 21, 2019.

[7] Anderson G , Robert Wood Johnson Foundation . Care Chronic: Making the Case for Ongoing Care. Chronic Care: Making the Case for Ongoing Care. https://www.rwjf.org/en/library/research/2010/01/chronic‐care.html. Published February 2010. Accessed January 27, 2020.

[8] Institute of Medicine (US) Committee on Quality of Health Care in America . Executive Summary ‐ Crossing the Quality Chasm ‐ NCBI Bookshelf. 2001.

[9] Sequist TD , Schneider EC , Anastario M , et al. Quality monitoring of physicians: linking patients’ experiences of care to clinical quality and outcomes. J Gen Intern Med. 2008;23(11):1784‐1790. 10.1007/s11606-008-0760-4 18752026 PMC2585686

[10] Doyle C , Lennox L , Bell D . A systematic review of evidence on the links between patient experience and clinical safety and effectiveness. BMJ Open. 2013;3(1):e001570 10.1136/bmjopen-2012-001570 PMC3549241 23293244

[11] Garg R , Shen C , Sambamoorthi N , Kelly K , Sambamoorthi U . Type of multimorbidity and patient‐doctor communication and trust among elderly medicare beneficiaries. Int J Family Med. 2016;2016:8747891 10.1155/2016/8747891 27800181 PMC5069353

[12] Levit L , Balogh E , Nass S , et al. Patient‐Centered Communication and Shared Decision Making ‐ Delivering High‐Quality Cancer Care ‐ NCBI Bookshelf. December 2013.

[13] Mollica MA , Enewold LR , Lines LM , et al. Examining colorectal cancer survivors’ surveillance patterns and experiences of care: a SEER‐CAHPS study. Cancer Causes Control. 2017;28(10):1133‐1141. 10.1007/s10552-017-0947-2 28866818

[14] Kinsella N , Stattin P , Cahill D , et al. Factors influencing Men’s choice of and adherence to active surveillance for low‐risk prostate cancer: a mixed‐method systematic review. Eur Urol. 2018;74(3):261‐280. 10.1016/j.eururo.2018.02.026 29598981 PMC6198662

[15] Loeb S , Walter D , Curnyn C , Gold HT , Lepor H , Makarov DV . How Active is Active Surveillance? Intensity of Followup during Active Surveillance for Prostate Cancer in the United States. J Urol. 2016;196(3):721‐726. 10.1016/j.juro.2016.02.2963 26946161 PMC5010531

[16] Butler SS , Loeb S , Cole AP , et al. United States trends in active surveillance or watchful waiting across patient socioeconomic status from 2010 to 2015. Prostate Cancer Prostatic Dis. 2020;23(1):179‐183. 10.1038/s41391-019-0175-9 31591454 PMC7202379

[17] Modi PK , Kaufman SR , Qi JI , et al. National trends in active surveillance for prostate cancer: validation of medicare claims‐based algorithms. Urology. 2018;120:96‐102. 10.1016/j.urology.2018.06.037 29990573 PMC6462187

[18] Chawla N , Urato M , Ambs A , et al. Unveiling SEER‐CAHPS®: a new data resource for quality of care research. J Gen Intern Med. 2015;30(5):641‐650. 10.1007/s11606-014-3162-9 25586868 PMC4395616

[19] Zaslavsky AM , Ayanian JZ , Zaborski LB . The validity of race and ethnicity in enrollment data for Medicare beneficiaries. Health Serv Res. 2012;47(3 Pt 2):1300‐1321. 10.1111/j.1475-6773.2012.01411.x 22515953 PMC3349013

[20] Health Resource & Service Administration . Area Health Resources Files. Area Health Resources Files. https://data.hrsa.gov/topics/health‐workforce/ahrf. Published July 31, 2019. Accessed February 13, 2020.

[21] Goodman RA , Posner SF , Huang ES , Parekh AK , Koh HK . Defining and measuring chronic conditions: imperatives for research, policy, program, and practice. Prev Chronic Dis. 2013;10:E66 10.5888/pcd10.120239 23618546 PMC3652713

[22] Johnston MC , Crilly M , Black C , Prescott GJ , Mercer SW . Defining and measuring multimorbidity: a systematic review of systematic reviews. Eur J Public Health. 2019;29(1):182‐189. 10.1093/eurpub/cky098 29878097

[23] Daskivich TJ , Fan K‐H , Koyama T , et al. Effect of age, tumor risk, and comorbidity on competing risks for survival in a U.S. population‐based cohort of men with prostate cancer. Ann Intern Med. 2013;158(10):709‐717. 10.7326/0003-4819-158-10-201305210-00005 23689764 PMC3760479

[24] Daskivich TJ , Thomas I‐C , Luu M , et al. External validation of the prostate cancer specific comorbidity index: a claims based tool for the prediction of life expectancy in men with prostate cancer. J Urol. 2019;202(3):518‐524. 10.1097/JU.0000000000000287 31009286

[25] Klabunde CN , Potosky AL , Legler JM , Warren JL . Development of a comorbidity index using physician claims data. J Clin Epidemiol. 2000;53(12):1258‐1267. 10.1016/s0895-4356(00)00256-0 11146273

[26] Klinkman MS . Competing demands in psychosocial care. A model for the identification and treatment of depressive disorders in primary care. Gen Hosp Psychiatry. 1997;19(2):98‐111. 10.1016/s0163-8343(96)00145-4 9097064

[27] National Cancer Institute . Case‐Mix Adjustment Guidance. Guidance on Standard Covariate Adjustment for SEER‐CAHPS Analyses. 2019 https://healthcaredelivery.cancer.gov/seer‐cahps/researchers/adjustment_guidance.html. Published November 4, 2019. Accessed February 13, 2020.

[28] Li C . Little’s test of missing completely at random. Stata J. 2013;13(4):795‐809. 10.1177/1536867X1301300407

[29] Auffenberg GB , Lane BR , Linsell S , Cher ML , Miller DC . Practice‐ vs physician‐level variation in use of active surveillance for men with low‐risk prostate cancer: implications for collaborative quality improvement. JAMA Surg. 2017;152(10):978‐980. 10.1001/jamasurg.2017.1586 28636713 PMC5831460

[30] Lang MF , Tyson MD , Alvarez JR , et al. The Influence of Psychosocial Constructs on the Adherence to Active Surveillance for Localized Prostate Cancer in a Prospective, Population‐based Cohort. Urology. 2017;103:173‐178. 10.1016/j.urology.2016.12.063 28189554 PMC5410889

[31] Tyson MD , Graves AJ , O’Neil B , et al. Urologist‐level correlation in the use of observation for low‐ and high‐risk prostate cancer. JAMA Surg. 2017;152(1):27‐34. 10.1001/jamasurg.2016.2907 27653425

[32] Bekelman JE , Rumble RB , Chen RC , et al. Clinically localized prostate cancer: ASCO clinical practice guideline endorsement of an american urological association/american society for radiation oncology/society of urologic oncology guideline. J Clin Oncol. 2018;36(32):3251‐3258. 10.1200/JCO.18.00606 30183466

[33] Volk RJ , McFall SL , Cantor SB , et al. It“s not like you just had a heart attack”: decision‐making about active surveillance by men with localized prostate cancer. Psychooncology. 2014;23(4):467‐472. 10.1002/pon.3444 24243777 PMC3983844

[34] Le Y‐C , McFall SL , Byrd TL , et al. “Active Surveillance” an acceptable alternative?: a qualitative study of couples’ decision making about early‐stage, localized prostate cancer. Narrat Inq Bioeth. 2016;6(1):51‐61. 10.1353/nib.2016.0006 27346824 PMC5176358

[35] Latini DM , Hart SL , Knight SJ , et al. The relationship between anxiety and time to treatment for patients with prostate cancer on surveillance. J Urol. 2007;178(3 Pt 1):826‐831. 10.1016/j.juro.2007.05.039 17632144

[36] Orom H , Nelson CJ , Underwood W , Homish DL , Kapoor DA . Factors associated with emotional distress in newly diagnosed prostate cancer patients. Psychooncology. 2015;24(11):1416‐1422. 10.1002/pon.3751 25631163 PMC5549449

[37] Goh AC , Kowalkowski MA , Bailey DE , Kazer MW , Knight SJ , Latini DM . Perception of cancer and inconsistency in medical information are associated with decisional conflict: a pilot study of men with prostate cancer who undergo active surveillance. BJU Int. 2012;110(2 Pt 2):E50‐E56. 10.1111/j.1464-410X.2011.10791.x 22145791

[38] Womble PR , Montie JE , Ye Z , et al. Contemporary use of initial active surveillance among men in Michigan with low‐risk prostate cancer. Eur Urol. 2015;67(1):44‐50. 10.1016/j.eururo.2014.08.024 25159890

[39] Filson CP , Schroeck FR , Ye Z , Wei JT , Hollenbeck BK , Miller DC . Variation in use of active surveillance among men undergoing expectant treatment for early stage prostate cancer. J Urol. 2014;192(1):75‐80. 10.1016/j.juro.2014.01.105 24518783

[40] Löppenberg B , Friedlander DF , Krasnova A , et al. Variation in the use of active surveillance for low‐risk prostate cancer. Cancer. 2018;124(1):55‐64. 10.1002/cncr.30983 28902401

[41] Boehm K , Dell’Oglio P , Tian Z , et al. Comorbidity and age cannot explain variation in life expectancy associated with treatment of non‐metastatic prostate cancer. World J Urol. 2017;35(7):1031‐1036. 10.1007/s00345-016-1963-7 27796538

[42] Kent M , Vickers AJ . A systematic literature review of life expectancy prediction tools for patients with localized prostate cancer. J Urol. 2015;193(6):1938‐1942. 10.1016/j.juro.2014.11.082 25463998 PMC4502577

[43] Raval AD , Madhavan S , Mattes MD , Sambamoorthi U . Types of chronic conditions combinations and initial cancer treatment among elderly Medicare beneficiaries with localised prostate cancer. Int J Clin Pract. 2016;70(7):606‐618. 10.1111/ijcp.12838 27291866 PMC4927389

[44] Daniels N . Equity of access to health care: some conceptual and ethical issues. Milbank Mem Fund Q Health Soc. 1982;60(1):51 10.2307/3349700 7038534

[45] Kinsella N , Helleman J , Bruinsma S , et al. Active surveillance for prostate cancer: a systematic review of contemporary worldwide practices. Translational Andrology and Urology; Vol 7, No 1 (February 2018): Translational Andrology and Urology (Prostate Cancer Screening and Active Surveillance in the Western World). 2018. 10.21037/tau.2017.12.24 PMC5861285 29594023

[46] Ehdaie B , Assel M , Benfante N , Malhotra D , Vickers A . A systematic approach to discussing active surveillance with patients with low‐risk prostate cancer. Eur Urol. 2017;71(6):866‐871. 10.1016/j.eururo.2016.12.026 28129893 PMC5714298

[47] Xu J , Neale AV , Dailey RK , Eggly S , Schwartz KL . Patient perspective on watchful waiting/active surveillance for localized prostate cancer. J Am Board Fam Med. 2012;25(6):763‐770. 10.3122/jabfm.2012.06.120128 23136314 PMC4212643

[48] Ennis RD , Parikh AB , Sanderson M , Liu M , Isola L . Interpreting oncology care model data to drive value‐based care: a prostate cancer analysis. J Oncol Pract. 2019;15(3):e238‐e246. 10.1200/JOP.18.00336 30742551

[49] D’Amico TA , Bandini LAM , Balch A , et al. Quality measurement in cancer care: a review and endorsement of high‐impact measures and concepts. J Natl Compr Canc Netw. 2020;18(3):250‐259. 10.6004/jnccn.2020.7536 32135508

[50] Center for Disease Control and Prevention . Health in All Policies | AD for Policy and Strategy | CDC. 2015 https://www.cdc.gov/policy/hiap/index.html. Published October 15, 2015. Accessed June 8, 2020.

