# Supplementary material for: Associations of multimorbidity and patient‐reported experiences of care with conservative management among elderly patients with localized prostate cancer
Source: Cancer Med. 2020 Jul 6;9(16):6051–61. doi: 10.1002/cam4.3274 (PMC7433828; doi:10.1002/cam4.3274)
Supplement: Supplementary file 2 — Table S2 [file CAM4-9-6051-s002.docx]

| **Supplemental Table 2. Multivariable Analysis of Timeliness of Care, Prostate Cancer Comorbidity Index, and Factors Associated with Conservative Management Use versus Treatment up to 24 Months after Incident Prostate Cancer Diagnosis among Fee-for-Service Medicare Beneficiaries using Linked SEER Cancer Registry with MCAHPS, 2002-2013 (n = 496).** | | | | | | | |
| --- | --- | --- | --- | --- | --- | --- | --- |
|  | | **UOR [95% CI]** | | | **AOR [95% CI]** | | **p-value** |
| **Multimorbidity** **Model** | | | | | | | |
| **Multimorbidity** | |  |  |  |  |  |  |
|  | Yes | 0.52 | [0.35 - 0.77] | 0.001 | 0.42 | [0.27 - 0.65] | <0.001 |
|  | No (Ref.) |  |  |  |  |  |  |
| **Getting Care Quickly** | | 1.13 | [1.02 - 1.24] | 0.014 | 1.21 | [1.09 - 1.35] | <0.001 |
| **Low-risk prostate cancer** | |  |  |  |  |  |  |
|  | Yes | 1.46 | [0.97 - 2.18] | 0.068 | 1.76 | [1.14 - 2.72] | 0.01 |
|  | No (Ref.) |  |  |  |  |  |  |
| **Mental Health** | |  |  |  |  |  |  |
|  | Fair/Poor | 3.44 | [1.73 - 6.81] | <0.001 | 4.32 | [1.86 - 10.1] | <0.001 |
|  | Excellent/Very Good (Ref.) |  |  |  |  |  |  |
| **Education** | |  |  |  |  |  |  |
|  | College or more | 2.76 | [1.35 – 5.65] | 0.006 | 3.21 | [1.50 – 6.89] | 0.003 |
|  | High-school graduate | 3.06 | [1.42 – 6.63] | 0.004 | 3.53 | [1.59-7.83] | 0.002 |
|  | No high-school graduation (Ref.) |  |  |  |  |  |  |
| **PCCI Model** | | | | | | | |
| **Prostate Cancer Comorbidity Index** | |  |  |  |  |  |  |
|  | 5 to 10 years life expectancy | 0.52 | [0.33 – 0.82] | 0.005 | 0.39 | [0.30 - 0.70] | <0.001 |
|  | < 5 years life expectancy | 0.56 | [0.31 – 1.01] | 0.056 | 0.34 | [0.16 – 0.73] | 0.005 |
|  | >10 years life expectancy (Ref.) |  |  |  |  |  |  |
| **Getting Care Quickly** | |  |  |  | 1.20 | [1.08 - 1.34] | 0.001 |
| **Low-risk prostate cancer** | |  |  |  | 1.76 | [1.11 – 2.79] | 0.017 |
|  | Yes |  |  |  |  |  |  |
|  | No (Ref.) |  |  |  |  |  |  |
| **Mental Health** | |  |  |  |  |  |  |
|  | Fair/Poor |  |  |  | 7.59 | [3.07 – 18.8] | <0.001 |
|  | Excellent/Very Good (Ref.) |  |  |  |  |  |  |
| **Education** | |  |  |  |  |  |  |
|  | High-school graduate |  |  |  | 0.26 | [0.11 – 0.60] | 0.002 |
|  | No high-school grad. (Ref.) |  |  |  |  |  |  |

Note: Based on 496 older (age >66 years) Fee-for-Service Medicare beneficiaries, with continuous enrollment in Medicare part A & Part B, diagnosed with incident localized prostate cancer between 2003 and 2013. Adjusted for age, race, marital status, income, education, health status, urologist density, radiation oncologist density, SEER region, geography, diagnostic year, and low-risk prostate cancer.

UOR= Unadjusted Odds Ratio, AOR= Adjusted Odds Ratio, N.S.= Not significant, CI= Confidence interval, Ref= Reference group, SEER= Surveillance, Epidemiology and End Results cancer Registry, MCAHPS= Medicare Consumer Assessment of Healthcare Providers and System surveys, CM= Conservative management, PCCI= Prostate Cancer Comorbidity Index. Statistically significant results displayed.
