# Supplementary material for: Associations of multimorbidity and patient‐reported experiences of care with conservative management among elderly patients with localized prostate cancer
Source: Cancer Med. 2020 Jul 6;9(16):6051–61. doi: 10.1002/cam4.3274 (PMC7433828; doi:10.1002/cam4.3274)
Supplement: Supplementary file 3 — Table S3 [file CAM4-9-6051-s003.docx]

| **Supplemental Table 3. Prostate cancer treatment codes used within 12 months of prostate cancer diagnosis to Identify Conservative Management Use** | |
| --- | --- |
| **Radical prostatectomy** | 55840, 55842, 55845, 55866, 55810, 55812, 55815 and 60.62, 17.42, 60.5, 60.4, and 60.3 (40.3, 40.53, and 40.59 for lymph node dissection) |
| **Brachytherapy** | 76873, 55859, 76965, 55860, 55875, 55876, 76873, 76965, 77326, 77327, 77328, 77761, 77762, 77763, 77799, 77776, 77777, 77778, and 60.99, 92.27, 92.28, and 92.29, 77781, 77782, 77783, 77784, 77785, 77786, 77787, 77789, 77790, Q3001, A9527, C1715, C1716, C1717, C1719, C1728, C2616, C2634, C2635, C2636, C2637, C2638, C2639, C2640, C2641, C2642, C2643, C2698, C2699, C9725 |
| **External Beam Radiotherapy** | 77305, 77310, 77315, 77321, 77371,77372, 77373, 77402, 77403, 77404, 77406, 77407, 77408, 77409, 77411, 77412, 77413, 77414, 77416, 77422, 77423, 92.24, 92.26, 77301, 77418, 0073T, 77380, 77381, 77520, 77522, 77523, and 77525, 77301,77338, 77761, 77762, 77763, 77789, 77427 |
| **Cryotherapy** | 50250, 50593, 55873, 55873, 60.62, C2618, G0160, G0161 |
| **Androgen Deprivation Therapy** | J1050, J1051 (Progesterone),J1950 J9217 J9218 J9219, C9430 (Lupron), J9165 (DES), J9202 (Zoladex), J3315 (Trelstar), J9225 CPT 11981(Vantas), all injections ICD-99.24, S0175 (Flutamide), J8999 (Bicalutamide/Flutamide/Nilutamide) S0165, J0128, C9216, (Abarelix), J9155 (Degarelix), S9560 (any hormone/adt), G0356 (any ADT ) |
| **Conservative management** | No treatment within one year of prostate cancer diagnosis |
